# Serine Protease 3 Promotes Progression of Diffuse Large B-Cell Lymphoma and Serves as a Novel Prognostic Predictor

**DOI:** 10.1155/2022/1254790

**Published:** 2022-12-30

**Authors:** Liangda Zheng, Xianting Wang, Weizhi Zheng, Hui Huang

**Affiliations:** Department of Hematology & Oncology, Taizhou First People's Hospital, Taizhou City 318020, China

## Abstract

Diffuse large B-cell lymphoma (DLBCL) ranks among the most prevalent malignancies of the lymphohematopoietic system in adults. The PRSS (Serine Protease) protein family members had been reported to be involved in carcinogenesis as well as tumor progression. Here, we aimed to explore the expression profile of PRSS3 in DLBCL and investigate its clinical significance as well as detailed functions. We retrospectively enrolled 155 DLBCL patients from our hospital and tested protein expression level of PRSS3 through immunohistochemical staining. Accordingly, PRSS3 was highly expressed in certain DLBCL tissues. Chi-square test revealed that higher PRSS3 expression was correlated with advanced Ann Arbor stage, elevated serum LDH level, and higher International Prognostic Index. Moreover, univariate and multivariate analyses confirmed that higher PRSS3 can act as an independent unfavorable prognostic predictor for DLBCL. Two human DLBCL cell lines, SUDHL10 and OCI-LY3, were subjected for knockdown assays, followed by phonotype tests including proliferation and invasion. According to the cellular experiments, PRSS3-knockdown resulted in impaired DLBCL proliferation in the two cell lines above. Taken together, PRSS3 is a novel prognostic factor for DLBCL, which functions by multiple signaling pathways.

## 1. Introduction

Diffuse large B-cell lymphoma (DLBCL) is the most common cancer in lymphohematopoietic system among adults, comprising about 30-40% of non-Hodgkin lymphoma [1]. In the past decades, there have been remarkable achievements in the diagnosis and treatment targeting DLBCL [2]. Nevertheless, the 5-year overall survival rate is only 60% due to its high heterogeneity and incurable with first-line treatment [3]. Therefore, more specific and sensitive clinical biomarkers and underlying mechanisms of DLBCL remain further investigation.

Trypsinogen family contains three protein members, which are encoded by *PRSS1*, *PRSS2*, and *PRSS3* genes, respectively [4]. Among them, mesotrypsin is encoded by the *PRSS3* gene, which is predominately produced and secreted from the human pancreas [5]. Interestingly, PRSS3 has been regarded to play critical roles in malignancies. For example, abnormal high expression of PRSS3 has been reported in non-small-cell lung cancer [6], pancreatic cancer [7], prostate cancer [8], etc. Furthermore, elevated PRSS3 was correlated with worse prognosis of certain tumor types such as pancreatic cancer [7] and prostate cancer [8]. Therefore, targeting PRSS3 may represent a potential intervention strategy in tumor treatment. However, its function and mechanisms in more malignancies are currently unknown.

Here, we initially mapped the mRNA and protein expression levels of PRSS3 in DLBCL tissue samples. As a result, elevated PRSS3 in DLBCL is correlated with unfavorable overall survival. In addition, we identified its oncogenic role in multiple DLBCL cell lines via knockdown strategy.

## 2. Methods

### 2.1. Online Database Mining

The mRNA level of DLBCL was extracted from TCGA datasets. In addition, we used UALCAN online server to further conduct Kaplan-Meier survival analysis [9].

### 2.2. Patients and Samples

Totally, 155 DLBCL tissues were acquired from Taizhou First People's Hospital. All tissue specimens were formalin-fixed and paraffin-embedded (FFPE). All diagnoses were based on pathological test. Patients with previously other malignancies were excluded.

### 2.3. Immunohistochemically Staining (IHC)

IHC was conducted as previously described [10]. Briefly, FFPE tissue samples were cut into 6 *μ*m sections, dried, deparaffinized, antigen retrieval, and blocked. Then section slides were incubated with primary PRSS3 antibody (MA5-24156, Thermo Fisher Scientific, Pittsburgh, PA) at 1 : 200 dilutions in 4°C overnight. On the second day, slides were rinsed and incubated with secondary antibody at room temperature for 30 minutes. Finally, slides were stained with 3,3 Diaminobenzidine (DAB) reaction buffer. IHC staining score was evaluated by multiplying the staining intensity (scored 0, 1, 2, and 3) and percentage of positively stained cells. According to the median IHC score, enrolled DLBCL cases were grouped into low-PRSS3 group (*n* = 74) or high-PRSS3 group (*n* = 81).

### 2.4. Primary Cell Isolation and Culture

Primary DLBCL cells were extracted and isolated from clinical obtained tumor samples. Briefly, specimens were washed with PBS, mechanically dissociated, filtered with 75 *μ*m nylon cell strainer, and suspended in single-cell status. Cells were then maintained in RPMI 1640 medium supplemented with 10% fetal bovine serum (FBS).

### 2.5. Cell Lines and shRNA Knockout

GCB and ABC cell lines were obtained from American Tissue Culture Collection (ATCC). We collected three GCB cell lines (SUDHL4, SUDHL6, and SUDHL10) and three ABC cell lines (TMD8, OCI-LY3, and OCI-LY10), respectively. All cells were cultured in RPMI 1640 medium containing 10% FBS.

Short hairpin RNA- (shRNA-) targeting PRSS3 was synthesized by GeneChem (Shanghai, China) into lentivirus using negative vector (vector) as control. The GV248 vector containing the sequence (TGAGCTTTGGTGCTGACTA) was constructed to perform PRSS3-silenced expression. Briefly, concentrated lentivirus was used to infect SUDHL10 and OCI-LY3 cells under the assistance of polybrene (10 *μ*g/mL). The knockdown efficiency was confirmed by western blot analyses.

### 2.6. Western Blot

Protein expression levels were evaluated with immunoblotting method [11]. Cultured cells were lysed using lysis buffer containing phosphatase inhibitor and protease inhibitor cocktail. After SDS-PAGE electrophoresis, extracted proteins were transferred to polyvinylidene difluoride (PVDF) membranes. Then, blotting membranes were blocked and incubated with primary antibodies at 1 : 1000 dilution (LS-B11527, LSBio, Seattle, WA) at 4°C overnight, followed by incubation with HRP-conjugated secondary antibodies for another 1 hour at 25°C. Finally, immunoblotting results were visualized using chemiluminescent (ECL) reagent. Each experiment was repeated three times.

### 2.7. Cell Proliferation Assay

MTT (3-[4, 5-dimethylthiazol-2-yl]-2, 5-diphenyl-tetrazolium) assay was conducted to evaluate cell proliferation capacity [12]. Briefly, transfected cells were seeded in 96-well plates at a density of 3000 cells/well. At every 24 hours later, 20 *μ*L MTT solution (5 mg/mL) was added into each well followed by incubating for 4 hours. Then removed the medium and 150 *μ*L dimethyl sulfoxide reagent was added to fully solubilize the MTT crystals. The absorbance was measured at 570 nm wavelength under a microplate reader.

### 2.8. Statistics

The SPSS 22.0 software was used for statistical analyses. During experimental assays, significance was evaluated by Student's *t*-test for two-group comparisons or one-way ANOVA for multiple comparisons. Clinical data were assessed using Chi-square test, Kaplan-Meier test, and Cox hazard regression test [13]. Two tail *P* < 0.05 was considered statistically significant.

### 2.9. Ethics

The Research Ethics Committee of Taizhou First People's Hospital reviewed and approved all protocols involving human specimens. Written informed consent was obtained from each participant.

## 3. Results

### 3.1. High-PRSS3 mRNA Level Is Associated with Unfavorable Prognosis of DLBC

**Figure 1:**
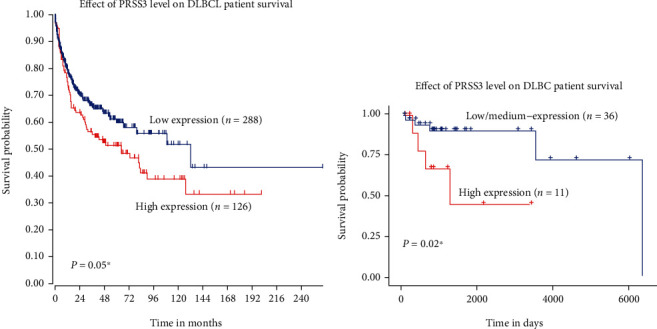
*PRSS3*-mRNA level was correlated with DLBCL prognosis. (a) TCGA dataset indicated that patients with higher *PRSS3*-mRNA level exhibited worse overall survival. (b) UALCAN online server analysis also showed that higher *PRSS3*-mRNA level was associated with worse DLBCL overall survival.

The mRNA expression data was extracted from TCGA dataset [14]. After dividing patients into low-PRSS3 group and high-PRSS3 group according to its mRNA level, Kaplan-Meier survival curve was plotted. As shown in Figure 1(a), patients with higher PRSS3 (*n* = 126) exhibited worse overall survival compared to those with lower PRSS3 (*n* = 288). Additionally, we assessed the mRNA level and clinical relevance of PRSS3 in another microarray set from UALCAN online server (Figure 1(b)). Accordingly, patients with elevated PRSS3 level exhibited shorter overall survival time than others (*P* = 0.002).

### 3.2. Enrolled Patients' Characteristics

The characteristics of enrolled patients (*n* = 155) were listed in Table 1. There were 63 females and 92 males enrolled. Among them, 73 patients were elder than 60 years at the time of diagnosis, while the other 82 cases were younger. The B symptoms were presented in 60 cases and absent in 95 cases. As for the Ann Arbor stage, 79 cases were staged with I-II, while the other 76 cases with III-IV. The ECOG PS was scored as 0-1 in 114 patients and scored larger than 1 in the other 41 patients. The serum LDH level was within normal range in 73 cases and was elevated in the other 82 cases. There were 102 patients suffered with 0-1 extra nodal involvement (ENI), while the other 53 cases with more than one ENI. Bulky tumor [15], as defined with tumors equal to or larger than 10.0 cm in diameter, was diagnosed in 35 cases. International Prognostic Index (IPI) score was 0-2 in 106 cases, while was scored 3-5 in 49 patients. According to the cell-of-origin (COO) molecular subtype, 71 cases were defined as germinal center B cell (GCB) subtype, while the other 84 patients with activated B cell (ABC) subtype.

### 3.3. PRSS3 Protein Expression Profile in DLBCL Tissues

**Figure 2:**
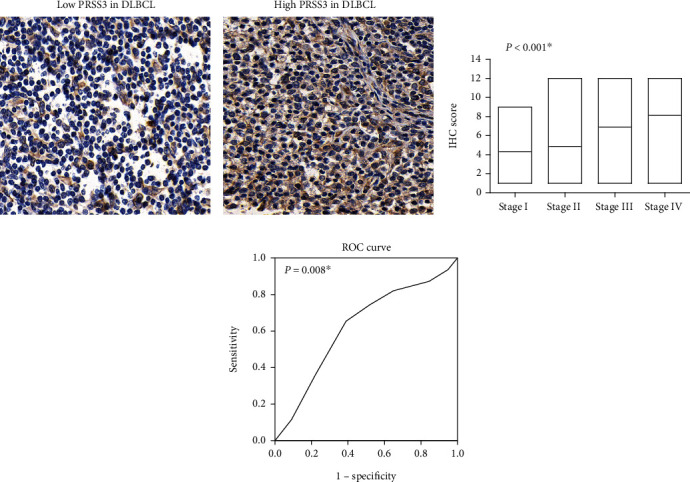
Protein expression of PRSS3 in DLBCL specimens. (a) Representative low-protein expression of PRSS3 in DLBCL tissues. (b) Representative high-protein expression of PRSS3 in DLBCL tissues. (c) PRSS3 IHC-staining scores in patients with different Ann Arbor stages were assessed and compared. (d) ROC curve was plotted to distinguish patients with high-PRSS3 expression or low-PRSS3 expression.

According to the IHC results of the 155 specimens above, PRSS3 protein predominantly expressed in the cytoplasm but with distinct expression level in different patients (Figures 2(a) and 2(b)). By evaluating its expression association with Ann Arbor stage, we found that higher PRSS3 IHC score was more frequently observed in higher stage patients (*P* < 0.001, Figure 2(c)). This finding encouraged us that PRSS3 may participate in DLBCL progression. Therefore, we divided patients into high-PRSS3 group (*n* = 81) and low-PRSS3 group (*n* = 74) according to the ROC method (Figure 2(d)).

cBesides Ann Arbor stage (*P* = 0.008), higher PRSS3 protein level was positively correlated with higher ECOG PS score (*P* = 0.002), elevated serum LDH level (*P* = 0.003), and higher IPI score (*P* < 0.001). All these clinicopathological factors had been reported to be correlated with DLBCL progression and unfavorable prognosis. Therefore, we next explored whether PRSS3 protein level has any effect on DLBCL patients' survival.

### 3.4. High-PRSS3 Protein Expression Indicates Worse Survival of DLBC

**Figure 3:**
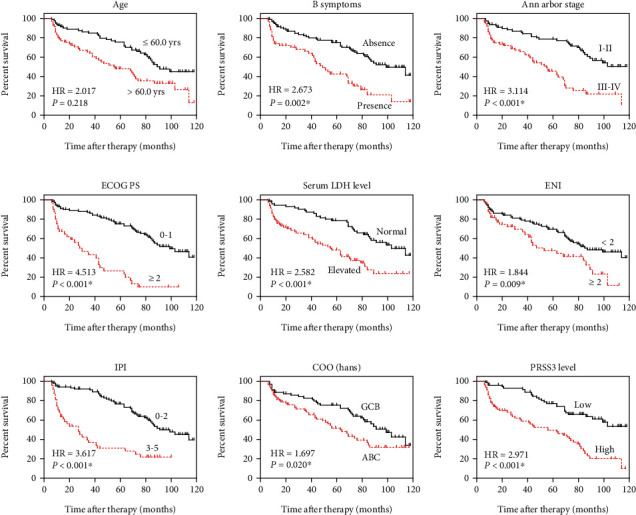
Survival analyses of enrolled DLBCL patients. The overall survival of enrolled DLBCL patients was analyzed in different subgroups according to patients age (a), B symptoms (b), Ann Arbor stage (c), ECOG PS (d), serum LDH level (e), ENI (f), IPI (g), COO (h), and PRSS3 protein levels (i). Data were analyzed using Kaplan-Meier method and compared with log-rank test. Abbreviations: PRSS3: serine protease 3; DLBCL: diffuse large B-cell lymphoma; ECOG PS: Eastern Cooperative Oncology Group performance status; LDH: lactate dehydrogenase; ENI: extra nodal involvement; IPI: International Prognostic Index; COO: cell-of-origin; GCB: germinal center B cell; and ABC: activated B cell.

We evaluated the prognostic effects of PRSS3 protein level and all retrieved clinicopathological variables using Kaplan-Meier method and log-rank test. Of note, although male patients exhibited worse overall survival than females (mean survival time 71.5 ± 4.4 vs. 78.5 ± 5.9 months), the difference was not statistically significant (*P* = 0.218, Table 2). As expected, younger patients showed a 23-month longer survival time than elder ones (Figure 3(a), *P* = 0.002). Also, positive B symptoms were correlated with worse 5-year survival rate (42.7% vs. 75.2%, Figure 3(b), *P* < 0.001). The 5-year survival rate of patients with Ann Arbor stage I-II was 78.6%, while decreased to 45.5% in those with stage III-IV (*P* < 0.001, Figure 3(c)). Patients with higher EOGC PS also exhibited lower 5-year survival rate (26.7% vs. 75.2%, *P* < 0.001, Figure 3(d)). Similarly, an elevated serum LHD level was correlated with unfavorable overall survival (*P* < 0.001, Figure 3(e)). More extra nodal involvement number also indicated shorter overall survival time (58.7 ± 5.9 months) compared to those with less ENI (80.0 ± 4.2 months, *P* = 0.009, Figure 3(f)). Patients with higher IPI index exhibited significantly lower 5-year survival rate (31.1% vs. 76.6%, *P* < 0.001, Figure 3(g)). Interestingly, patients with GCB molecular type had better overall survival than those with ABC molecular type (mean survival time 83.1 ± 4.8 vs. 65.2 ± 5.0 months, *P* = 0.020, Figure 3(h)).

For the first time, our data identified that higher PRSS3 protein expression level was correlated with unfavorable overall survival of DLBCL (*P* < 0.001, Figure 3(i)). In detail, the mean survival time of low-PRSS3 group was 90.9 ± 4.3 months with a 5-year overall survival rate 77.2%, while the mean survival time of higher PRSS3 group was 57.0 ± 4.9 months with a 5-year overall survival rate 49.4%.

Moreover, we conducted multivariate analysis using a Cox hazard regression model (Table 3). Accordingly, higher PRSS3 protein level was identified as a novel independent prognostic factor of DLBCL (hazard ratio 1.8, 95% confidence interval 1.04-3.14, *P* = 0.036). In our cohort, other independent risk factors included elder age, B symptoms, advanced Ann Arbor stage, higher ECOG PS, elevated serum LDH level, higher IPI score, and ABC molecular type (all *P* < 0.05).

### 3.5. Silencing PRSS3 Results in Decreased DLBCL Proliferative Capacity

**Figure 4:**
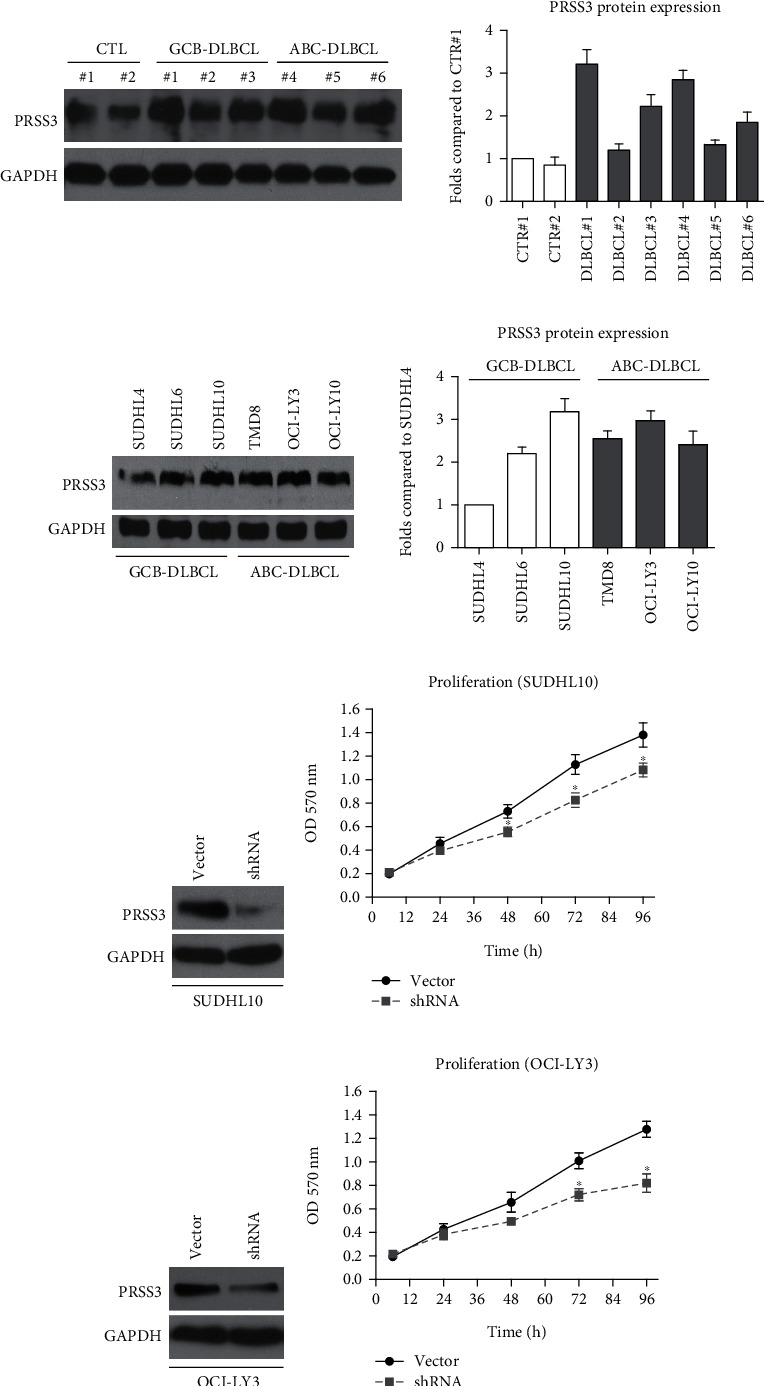
Silencing PRSS3 impaired DLBCL proliferation. (a) Protein expression level of PRSS3 was tested and compared in primary cells generated from different clinical specimens. (b) Protein expression level of PRSS3 was tested and compared in different commercially available DLBCL cell lines. (c) SUDHL10 cells were treated with PRSS3-shRNA or control-shRNA. The knockdown effectiveness was evaluated by immunoblotting. (d) SUDHL10 cell proliferation curves were plotted according to the MTT assay. (e) OCI-LY3 cells were treated with PRSS3-shRNA or control-shRNA. The knockdown efficiency was evaluated by immunoblotting. (f) OCI-LY3 cell proliferation curves were plotted according to the MTT assay.

Since clinical data indicated that PRSS3 may play critical roles in DLBCL progression, we next performed cellular experiments to explore its detailed functions. By testing its protein level in primary cells, we confirmed that PRSS3 was indeed upregulated in DLBCL cells (Figure 4(a)). Additionally, we collected three DLBCL cell lines with GCB molecular type and another three DLBCL cell lines with ABC molecular type. Immunoblotting data showed diverse PRSS3 protein levels in different cell lines; nevertheless, no significant difference was identified within the two molecular types (Figure 4(b)).

It is well-acknowledged that DLBCL with GCB or ABC type possesses completely different molecular profiles; therefore, we conducted shRNA-knockdown experiments in both types. Among the six cell lines, SUDHL10 and OCI-LY3 were selected due to their highest endogenous PRSS3 levels in GCB and ABC types, respectively. As a result, silencing PRSS3 in SUDHL10 cells significantly inhibited cell proliferation as reflected by MTT assays (Figures 4(c) and 4(d)). Similarly, shRNA-directed knockdown of PRSS3 in OCI-LY3 cells led to decreased proliferative capacity of OCI-LY3 cells (Figures 4(e) and 4(f)).

## 4. Discussion

The expression and function of PRSS3 in different cancers are completely different. On one hand, PRSS3 is decreased in certain cancer types and may play tumor-suppressing roles. For example, although HPV infection is a well-known pathogen for squamous cell carcinoma of the head and neck, its infection may induce a decreased PRSS3 level in this cancer type [16]. Similarly, methylation of PRSS3 was identified in 53% (86 of 166) of lung cancers examined by Marsit et al. [17], which may result in decreased PRSS3 expression. Furthermore, Lin et al. reported that PRSS3 was decreased in hepatocellular carcinoma (HCC) and PRSS3 overexpression inhibited HCC cell cycle, proliferation, migration, and invasion [18]. Their data demonstrated a tumor-suppressing role of PRSS3 in HCC via multiple pathways, including downregulation of matrix metallopeptidase 2 (MMP2) and deactivating MEK1-ERK1/2 signaling [18].

On the other hand, PRSS3 may exert oncogenic functions in more cancer types. Here in the current study, we firstly reported an upregulated expression of PRSS3 and elucidated its prognostic value as well as tumor-promoting effects in DLBCL. Based on our results, silencing PRSS3 can significantly attenuate DLBCL growth in two DLBCL cell lines. Consistent with our data, knockdown of PRSS3 attenuates, while stimulation with recombinant purified mesotrypsin enhances, the proliferation of breast cancer [19]. Interestingly, PRSS3 was only observed to be overexpressed in metastatic pancreatic cancer cells but not in nonmetastatic pancreatic cancer cells [7]. Their findings indicated that PRSS3 may participate in tumor metastasis and their further data confirmed a significant correlation between PRSS3 expression and metastasis in clinical samples. Similar findings were observed in several other cancer types on that PRSS3 overexpression could serve as a survival predictor of ovarian cancer, gastric cancer, and colon cancer [20–22].

As for the detailed upstream and downstream signaling pathways, Jiang et al. demonstrated that PRSS3 upregulated VEGF expression via the PAR1-mediated ERK pathway in pancreatic cancer [7]. According to their data, ERK inhibitor significantly attenuated pancreatic cancer progression and prolonged the survival time of mice model [7]. Besides, Hockla et al. suggested CD109 as the functional proteolytic target of mesotrypsin in breast cancer using mass-spectrometry method [19]. Another reported downstream substrate of PRSS3 is the Tissue Factor Pathway Inhibitor-2 (TFPI-2). Ghilardi et al. implied that TFPI-2 can directly interact with and was degraded by active PRSS3, thus promoting of tumor vascular migration [23]. Expression of PRSS3 can be modulated by methylation regulation [24] and upstream microRNAs such as miR-217 [25]. Whether these signaling pathways are involved in DLBCL remain further investigations.

Previously, Hockla et al. demonstrated that PRSS3 was upregulated in metastatic prostate cancer tissues and recombinant mesotrypsin facilitating an invasive cellular phenotype in prostate cancer cells [8]. Moreover, they showed the tumor-promoting role of PRSS3 depended on its enzymatic activities [8]. Therefore, its specific inhibitor could provide potential novel therapeutics directions. In the past decade, more and more groups were focusing on PRSS3 inhibitors and some of them developed promising results [26, 27]. For example, Salameh et al. demonstrate the efficacy of an improved PRSS3 inhibitor targeting breast cancer proliferation and pancreatic cancer invasion [28]. Another example is that targeting the PRSS3 in conjunction with current 5-Fu therapy provides further inhibition effects in esophageal adenocarcinoma [29]. Similarly, PRSS1, another PRSS3 protein family member, was reported to be negatively associated with the sensitivity of ovarian cancer cells to cetuximab, which functions by cleaving cetuximab thus leading to resistance [30]. Nevertheless, further evidence is required to obtain better selectivity and higher inhibition efficiency.

## 5. Conclusions

PRSS3 is upregulated in DLBCL and correlated with unfavorable clinical outcomes. Elevated PRSS3 enhances DLBCL proliferation while targeting PRSS3 can significantly suppress DLBCL progression.

## Figures and Tables

**Table 1:** Correlations between PRSS3 expression and DLBCL patients' features.

Variables	Cases	PRSS3 protein level		*P* value
	(*n* = 155)	Low (*n* = 74)	High (*n* = 81)	
Sex				0.763
Female	63	31	32	
Male	92	43	49	
Age				0.784
≤60.0 yrs	82	40	42	
>60.0 yrs	73	34	39	
B symptoms				0.125
Absence	95	50	45	
Presence	60	24	36	
Ann Arbor stage				0.008^∗^
I-II	79	46	33	
III-IV	76	28	48	
ECOG PS				0.002^∗^
0-1	114	63	51	
≥2	41	11	30	
Serum LDH level				0.003^∗^
Normal	73	44	29	
Elevated	82	30	52	
ENI				0.659
<2	102	50	52	
≥2	53	24	29	
Bulky tumor				0.785
No	120	58	62	
Yes	35	16	19	
IPI				<0.001^∗^
0-2	106	64	42	
3-5	49	10	39	
COO (Hans)				0.722
GCB	71	35	36	
ABC	84	39	45	

Abbreviations: PRSS3: serine protease 3; DLBCL: diffuse large B-cell lymphoma; ECOG PS: Eastern Cooperative Oncology Group performance status; LDH: lactate dehydrogenase; ENI: extra nodal involvement; IPI: International Prognostic Index; COO: cell-of-origin; GCB: germinal center B cell; ABC: activated B cell.

**Table 2:** Kaplan-Meier overall survival analyses.

Variables	Cases (*n* = 155)	OS months (mean ± S.D.)	5-year OS (%)	*P* value
Sex				
Female	63	78.5 ± 5.9	61.7%	0.218
Male	92	71.5 ± 4.4	64.3%	
Age				
≤60 yrs	82	84.4 ± 4.4	75.5%	0.002^∗^
>60 yrs	73	61.3 ± 5.4	48.1%	
B symptoms				
Absence	95	84.9 ± 4.2	75.2%	<0.001^∗^
Presence	60	55.0 ± 5.5	42.7%	
Ann Arbor stage				
I-II	79	88.9 ± 4.4	78.6%	<0.001^∗^
III-IV	76	56.1 ± 4.9	45.5%	
ECOG PS				
0-1	114	85.8 ± 3.7	75.2%	<0.001^∗^
≥2	41	38.1 ± 5.2	26.7%	
Serum LDH level				
Normal	73	89.1 ± 4.3	78.5%	<0.001^∗^
Elevated	82	58.7 ± 5.1	48.2%	
ENI				
<2	102	80.0 ± 4.2	69.6%	0.009^∗^
≥2	53	58.7 ± 5.9	47.6%	
Bulky tumor				
No	120	77.1 ± 3.9	66.8%	0.240
Yes	35	64.2 ± 8.3	51.4%	
IPI				
0-2	106	86.7 ± 3.6	76.6%	<0.001^∗^
3-5	49	41.0 ± 5.7	31.1%	
COO (Hans)				
GCB	71	83.1 ± 4.8	75.3%	0.020^∗^
ABC	84	65.2 ± 5.0	51.6%	
PRSS3 protein level				
Low	74	90.9 ± 4.3	77.2%	<0.001^∗^
High	81	57.0 ± 4.9	49.4%	

Abbreviations: PRSS3: serine protease 3; DLBCL: diffuse large B-cell lymphoma; ECOG PS: Eastern Cooperative Oncology Group performance status; LDH: lactate dehydrogenase; ENI: extra nodal involvement; IPI: International Prognostic Index; COO: cell-of-origin; GCB: germinal center B cell; ABC: activated B cell.

**Table 3:** Multivariate analysis.

Variables	HR	95% CI	*P* value
Age (>60 vs. ≤60 yrs)	1.778	1.101-2.871	0.019^∗^
B symptoms (presence vs. absence)	2.565	1.543-4.262	<0.001^∗^
Ann Arbor stage (III-IV vs. I-II)	2.520	1.505-4.218	<0.001^∗^
ECOG PS (≥2 vs. 0-1)	1.924	1.089-3.398	0.024^∗^
Serum LDH level (elevated vs. normal)	1.871	1.125-3.111	0.016^∗^
ENI (≥2 vs. <2)	1.435	0.854-2.411	0.172
IPI (3-5 vs. 0-2)	3.435	1.918-6.151	<0.001^∗^
COO (ABC vs. GCB)	1.699	1.047-2.757	0.032^∗^
PRSS3 protein level (high vs. low)	1.807	1.040-3.138	0.036^∗^

Abbreviations: PRSS3: serine protease 3; ECOG PS: Eastern Cooperative Oncology Group performance status; LDH: lactate dehydrogenase; ENI: extra nodal involvement; IPI: International Prognostic Index; COO: cell-of-origin; GCB: germinal center B cell; ABC: activated B cell.

## Data Availability

The data will be available upon rational request.
